# Prioritizing Therapeutics for Lung Cancer: An Integrative Meta-analysis of Cancer Gene Signatures and Chemogenomic Data

**DOI:** 10.1371/journal.pcbi.1004068

**Published:** 2015-03-18

**Authors:** Kristen Fortney, Joshua Griesman, Max Kotlyar, Chiara Pastrello, Marc Angeli, Ming Sound-Tsao, Igor Jurisica

**Affiliations:** 1 Department of Medical Biophysics, University of Toronto, Toronto, Ontario, Canada; 2 Princess Margaret Cancer Centre, University Health Network, Toronto, Ontario, Canada; 3 Department of Computer Science, University of Toronto, Toronto, Ontario, Canada; 4 Techna Institute, University Health Network, Toronto, Ontario, Canada; National University of Singapore, SINGAPORE

## Abstract

Repurposing FDA-approved drugs with the aid of gene signatures of disease can accelerate the development of new therapeutics. A major challenge to developing reliable drug predictions is heterogeneity. Different gene signatures of the same disease or drug treatment often show poor overlap across studies, as a consequence of both biological and technical variability, and this can affect the quality and reproducibility of computational drug predictions. Existing algorithms for signature-based drug repurposing use only individual signatures as input. But for many diseases, there are dozens of signatures in the public domain. Methods that exploit all available transcriptional knowledge on a disease should produce improved drug predictions. Here, we adapt an established meta-analysis framework to address the problem of drug repurposing using an ensemble of disease signatures. Our computational pipeline takes as input a collection of disease signatures, and outputs a list of drugs predicted to consistently reverse pathological gene changes. We apply our method to conduct the largest and most systematic repurposing study on lung cancer transcriptomes, using 21 signatures. We show that scaling up transcriptional knowledge significantly increases the reproducibility of top drug hits, from 44% to 78%. We extensively characterize drug hits *in silico*, demonstrating that they slow growth significantly in nine lung cancer cell lines from the NCI-60 collection, and identify CALM1 and PLA2G4A as promising drug targets for lung cancer. Our meta-analysis pipeline is general, and applicable to any disease context; it can be applied to improve the results of signature-based drug repurposing by leveraging the large number of disease signatures in the public domain.

## Author Summary

Computer algorithms that find new uses for known drugs can accelerate the development of new therapies for many diseases, including cancer. One promising strategy is to identify drugs that, at the transcriptional level, reverse the gene expression signature of a disease. A major difficulty with this strategy is variability: different gene expression signatures of the same disease or drug treatment can show poor overlap across studies. Since existing algorithms analyze one signature at a time, this means that the drug candidates they identify may reverse some signatures of a disease but not others. For many diseases, dozens of signatures from different labs are now available in online databases. Combining knowledge across all signatures should lead to better drug predictions. Here, we design a meta-analysis pipeline that takes in a large set of disease signatures and then identifies drugs that consistently reverse deleterious gene changes. We apply our method to find new drug candidates for lung cancer, using 21 signatures. We show that our meta-analysis pipeline increases the reproducibility of top drug hits, and then extensively characterize new lung cancer drug candidates *in silico*.

## Introduction

Lung cancer accounts for the largest number of cancer-related deaths, and the 5-year survival rate (across all stages) is only 16% [1]; there is an urgent need for new therapeutics to help treat it. Over the past two decades, the application of high-throughput technologies has led to the rapid accumulation of comprehensive and diverse public datasets cataloguing genome-wide molecular alterations seen with lung cancer or with drug administration. Integrative computational methods that mine these data are fast, cheap, and can complement traditional methods of drug screening; complementary information in these distinct resources can be leveraged to develop comprehensive *in silico* screens for novel cancer therapeutics [2].

One such resource, the Connectivity Map (CMap), which is the focus of our analyses, catalogues the transcriptional responses to drug treatment in human cell lines for over a thousand small molecules [3]. CMap has been successfully applied to identify novel therapeutics for a diverse set of indications including various cancers [4,5], and most recently osteoarthritic pain [6] and muscle atrophy [7].

CMap was applied in three earlier studies to identify novel therapeutics for lung cancer. Wang et al. [8] combined two microarray data sets to create a single transcriptional signature of lung adenocarcinoma and screened it against CMap. They tested one of their drug hits (17-AAG) *in vitro* and found that it inhibited growth in two lung adenocarcinoma cell lines. Ebi et al. [9] constructed a transcriptional signature of survival in patients with lung adenocarcinoma; CMap analysis identified several drugs that might improve outcome. The authors experimentally confirmed the growth inhibitory activity of several drug hits, including rapamycin, LY-294002, prochlorperazine, and resveratrol. Jahchan et al. [10] combined two public datasets on small cell lung cancer into a single signature and screened it against the drug profiles in CMap. *In vitro* experiments confirmed the inhibitory activity of many of their top hits, and *in vivo* testing showed promising results for imipramine and promethazine.

Nearly every previous analysis using Connectivity Map data to link drugs to diseases has done so with the CMap online tool (http://broadinstitute.org/cmap/). The CMap tool takes as input a set of up-regulated probe sets and a set of down-regulated probe sets, and returns a list of drugs that reverts or mimics those gene expression changes. However, for most diseases, not one but many—often dozens—of distinct gene signatures are available. For example, the cancer-specific database Oncomine (version 4.4) currently stores mRNA data from 566 different studies [11]. As the CMap tool only deals with one gene signature at a time, the question of how best to take advantage of the information in a large collection of disease signatures remains an important open problem. Since different disease signatures can overlap poorly from study to study [2], combining information across many signatures has the potential to improve the performance of drug repurposing algorithms.

While a few studies have used multiple disease signatures in CMap analysis, e.g., [7,8] (though with one exception [12], they used only two or three signatures), they have all relied on essentially the same strategy of collapsing all disease signatures into a single meta-signature (by e.g., intersecting lists of significant genes from different studies, as in [7]) and querying the CMap data with this signature. Since each of the individual disease signatures was constructed using dozens or even hundreds of microarrays, there is fairly strong evidence for every gene in each signature. In contrast, the drug response data in CMap is noisy: the 1,309 drugs have each been tested only a median of 4 times (4 treatment microarrays). This noise has consequences: previous work has shown that even small changes in the input gene signature can lead to large changes in the list of drugs identified as significant by CMap analysis (with the sscMap program) [13,14].

Here we propose an alternative strategy for connecting a set of disease gene signatures to drugs, CMapBatch. Rather than collapsing all the gene signatures in the set into a single gene signature, we propose to screen each disease signature separately against CMap to produce a set of ranked lists of drug candidates. Next, we apply meta-analysis to identify which drugs are consistently ranked as the best candidates across all disease signatures. Thus, we perform the meta-analysis at a later step: our method combines lists of drugs rather than lists of genes. We show that this strategy returns more stable sets of top drug candidates compared to when individual gene signatures are used.

Next, we applied CMapBatch to lung cancer. We used three steps to identify and prioritize new lung cancer therapeutics. First, we conducted a meta-analysis using CMapBatch to identify drugs that reverse the transcriptional changes seen with lung cancer across 21 gene signatures (see Table 1). We identified 247 CMap drugs that consistently counter the gene changes that occur with lung cancer. Second, we performed *in silico* validation of drug candidates with the NCI-60 growth inhibition data. This validation supported our method: drug candidates identified by CMapBatch were significantly more likely to slow growth in nine lung cancer cell lines than other CMap drugs. Third, we implemented data integration for drug prioritization. We identified common protein targets of significant drugs, and used chemical structure similarity and drug-target relationships to prioritize candidate therapeutics.

**Table 1:** Twenty-one lung cancer gene signatures (tumour vs. normal comparisons).

Histology	PMID	Source
**Adenocarcinoma**	18992152	CDIP
**Adenocarcinoma**	16549822	CDIP
**Adenocarcinoma**	18927117	CDIP
**Adenocarcinoma**	12118244	Oncomine
**Adenocarcinoma**	11707567	Oncomine
**Carcinoid**	11707567	Oncomine
**Small Cell**	11707567	Oncomine
**Squamous**	11707567	Oncomine
**Large Cell**	11707590	Oncomine
**Adenocarcinoma**	11707590	Oncomine
**Small Cell**	11707590	Oncomine
**Squamous**	11707590	Oncomine
**Large Cell**	20421987	Oncomine
**Adenocarcinoma**	20421987	Oncomine
**Squamous**	20421987	Oncomine
**Adenocarcinoma**	18297132	Oncomine
**Adenocarcinoma**	16314486	Oncomine
**Adenocarcinoma**	17540040	Oncomine
**Squamous**	15833835	Oncomine
**Squamous**	16188928	Oncomine
**Squamous**	14581339	Oncomine

## Results

### CMapBatch meta-analysis strategy: From individual cancer gene signatures to candidate therapeutics

**Fig 1:**
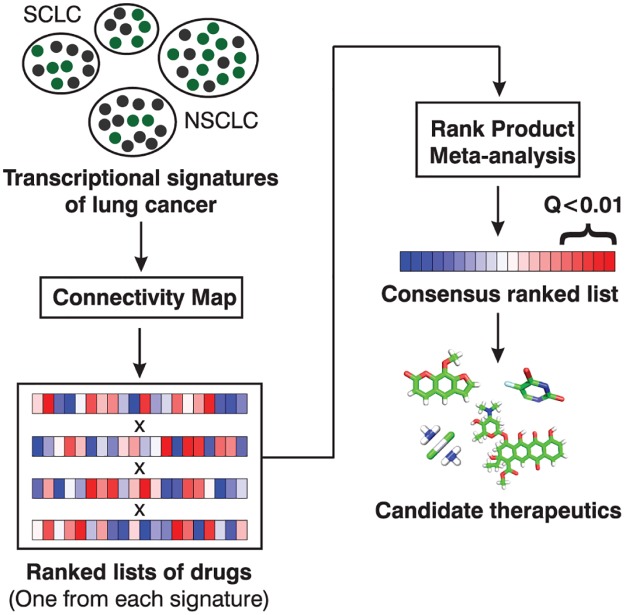
CMapBatch meta-analysis pipeline. Given a set of disease signatures, CMapBatch calculates mean connectivity scores for 1,309 drugs and converts them to ranks. Next, CMapBatch applies the Rank Product method to identify drugs that are consistently highly ranked across signatures. On a set of 21 transcriptional signatures of lung cancer, we identified 247 drugs that significantly reverse these pathological gene expression changes (at FDR < 1%).

Our CMapBatch meta-analysis pipeline comprises the following steps (Fig. 1): For each individual lung cancer signature (tumour vs. normal comparison), we calculate mean connectivity scores for 1,309 small molecules (as previously described [3]). Connectivity scores range between -1 and 1; a large, negative mean connectivity score indicates that drug treatment reverses many of the gene changes seen with lung cancer. We use the mean connectivity score to construct a ranked list of drugs for each signature. We combine the ranked lists of drugs into a single matrix, and identify drugs that were consistently highly ranked across all signatures using the Rank Product method [15] (see Materials and Methods).

Our analyses are based on 21 previously published gene expression signatures of lung cancer obtained from Oncomine [11] and CDIP, the Cancer Data Integration Portal (http://ophid.utoronto.ca/cdip/). The samples used to derive each signature have diverse histologies, and mRNA levels were measured on various commercial platforms.

### Candidate drugs identified via CMapBatch are more conserved across signature subsets than candidate drugs identified from single gene signatures

**Fig 2:**
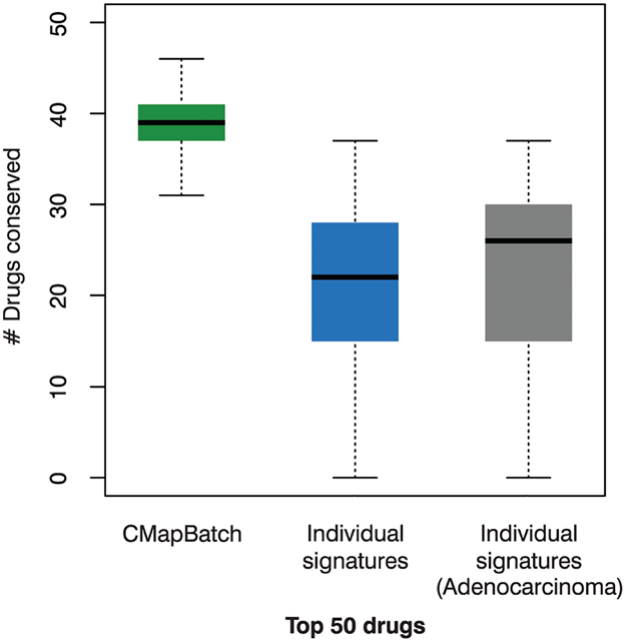
CMapBatch produces more stable lists of significant drugs than individual gene signatures. Shown are boxplots of the number of conserved drug candidates when any two lists of top 50 drug candidates are intersected. Green: 21 gene signatures were split into two disjoint sets of 10 and 11 signatures, CMapBatch was run on both sets, and top drugs from each set were compared; this experiment was repeated 100 times. Blue: 21 gene signatures were used to retrieve 21 lists of drugs with the CMap online tool; top drugs from all pairs of signatures were compared. Grey: 10 gene signatures of the same lung cancer type (adenocarcinoma) were used to retrieve 10 lists of drugs with the CMap online tool; top drugs from all pairs of signatures were compared. CMapBatch results showed a significantly higher median overlap (Wilcox test P << 0.01).

Previous work has shown that CMap analysis of different gene signatures for the same disease can return very different lists of drug candidates [14]. This is undesirable, if perhaps unsurprising as gene signatures themselves can be highly variable [2]. Consistent with previous findings, we found that when we retrieved lists of the top 50 drugs for each of the 21 different gene signatures of lung cancer (using the CMap online tool), overlap was poor. The median number of drug candidates present in top 50 drug candidate lists from two different signatures was only 22 (Fig. 2 in blue). Repeating the same test using lung cancer signatures of the same type—10 adenocarcinoma signatures—did not lead to much improvement. For adenocarcinoma, the median number of drugs identified by two signatures was 26 (Fig. 2 in gray), but the difference is not statistically significant. We also tested whether the signatures were heterogeneous by computing, for each signature, the median number of drugs shared with all other signatures. For 19 signatures, the median number of shared drugs between any pair of them was similar, varying from 16–29. But there were two outliers: an adenocarcinoma signature [16] that shares zero drugs with any other signature, and a signature of carcinoid tumours [17] that shares a median of only three drugs with other signatures. Removing these two heterogeneous signatures from the signature set boosts the median number of drugs common to any pair of signatures to 24, but again this difference is not statistically significant.

Next, we sought to determine whether aggregating the information from a large set of signatures with CMapBatch would lead to a more stable list of top drug candidates. For this test, we randomly assigned the 21 lung cancer gene signatures to two groups, one with 10 and the other with 11 signatures. We ran CMapBatch separately on the two disjoint sets of signatures, and compared lists of the top 50 drugs identified for each set. We repeated this test 100 times. We found that CMapBatch consistently identifies the same drugs as combatting lung cancer, even when it is trained on completely different sets of lung cancer signatures. A median of 39 drugs were found to be common to both the lists of top 50 drugs identified from two disjoint sets of signatures (Fig. 2 in green), significantly more than are found with individual gene signatures (Wilcox test P << 0.01). This key finding is not sensitive to choice of threshold; using the top 25 or top 100 drugs as an alternative cut-off, CMapBatch again recovers a significantly higher number of drugs (P << 0.01; S1 Fig).

### Characterizing and prioritizing candidate lung cancer therapeutics

For the remainder of this paper, we focus on characterizing and prioritizing the full set of significant drugs identified by CMapBatch using all 21 gene signatures of lung cancer.

#### CMapBatch meta-analysis identified 247 candidate lung cancer therapeutics

At an FDR cut-off of 0.01, we find that 247 drugs (out of 1,309 drugs in CMap Build 2) significantly reverse the gene expression changes seen with lung cancer in the full set of 21 lung cancer signatures (S1 Table). This is a large number of drugs, but in line with previous results obtained using similar data; e.g., a recent paper examining disease-drug relationships using the 164 drugs tested in CMap Build 1 linked 72 of them to adenocarcinoma of the lung, and 67 to squamous cell carcinoma of the lung [12].

### Candidate therapeutics inhibit growth in nine NSCLC cell lines

As an independent validation of our results, we used growth inhibition data from the NCI-60 collection [18] to determine whether the drug candidates we identified are better at slowing growth in lung cancer cell lines. For all our NCI-60 analyses we used the nine lung cancer cell lines in which over 100 Connectivity Map drugs were tested (see Methods). None of these nine cell lines were included in the CMap dataset, so they provide an independent test of the effectiveness of our predicted drugs for lung cancer.

#### Significant drugs are more effective at inhibiting growth than other Connectivity Map drugs

**Fig 3:**
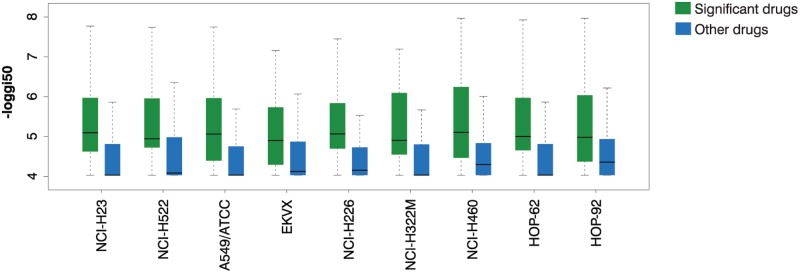
Drug candidates inhibit growth in lung cancer cell lines more than other Connectivity Map drugs. We tested whether the drugs that we identified as significantly reversing the gene changes seen with lung cancer were better at inhibiting growth using NCI-60 GI50 data in 9 lung cancer cell lines. In every cell line, significant drugs are better than other Connectivity Map drugs at inhibiting growth (Wilcox test P < 0.01). On the y axis, growth inhibition is presented as-log10 GI50, so that e.g. a value of 6 corresponds to a pGI50 of 10^-6^ M.

In all nine cell lines, drugs that CMapBatch identifies as reversing the transcriptional changes seen with lung cancer are significantly better than other CMap drugs at inhibiting growth (Wilcox test P < 0.01; Fig. 3). For example, in NCI-H23 lung adenocarcinoma cells, the median pGI50 for our predicted lung cancer drugs is 10^–5.1^ M, while for other CMap drugs it is 10^-4.0^ M (P < 10^-4^); values of 10^-4^ M are considered inactive in NCI-60. Using a stringent threshold of 5 μM for evidence of target-mediated drug behavior, 46% of our drug candidates show pGI50 values less than 5 μM, while only 23% of other CMap drugs achieve this, a two-fold difference.

#### 23 significant drugs inhibit growth in a majority of lung cancer cell lines

**Fig 4:**
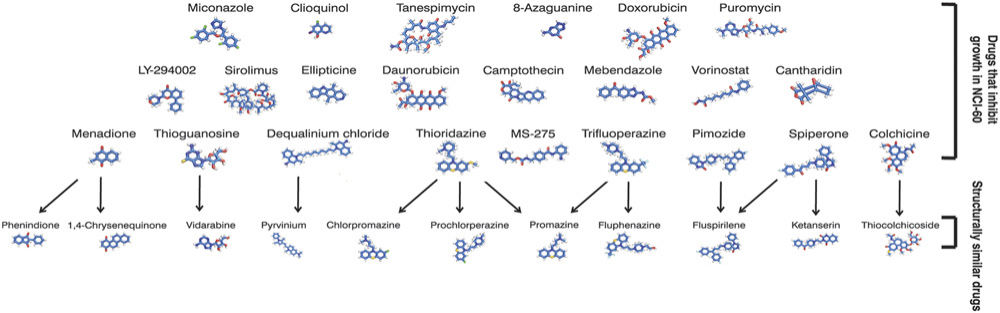
Prioritizing drug candidates with GI50 values and chemical structures. Twenty-three of the significant drugs inhibit growth in a majority of lung cancer cell lines (top). A further 11 significant drugs not tested in NCI-60 are highly structurally similar (Tanimoto similarity > = 0.8) to one or more of the sixteen (bottom).

For each of the nine cell lines, and using data from every drug tested on that line, we define the threshold for sensitivity to a drug to be the top 20% of the -logGI50 values; i.e., we say that the cell line is sensitive to those drugs with -logGI50 values in the top 20%. In practice, this corresponds to imposing a pGI50 threshold of 5.45 μM—16.83 μM, depending on the cell line. By this definition, of all the NCI60 drugs that have been tested in five or more lung cancer cell lines, 7,794 of 44,802, or 17%, inhibit growth in 5 or more cell lines. In total, 167 CMap drugs were tested in these NCI-60 lung cancer cell lines, including 41 drugs that we identified as significant in our meta-analysis. Of the significant drugs tested, 23/41, or 56% inhibit growth in 5 or more lung cancer cell lines (Fig. 4, left). Also, each of these 23 drugs shows a pGI50 < 5 μM in at least one cell line that we tested.

Among these 23 are several that are already in use to treat cancer. For example, daunorubicin and the chemically related doxorubicin are topoisomerase inhibitors and commonly-used chemotherapeutic agents; sirolimus (rapamycin) is currently in clinical trials for several cancers, and was recently shown to increase NSCLC tumour cell sensitivity to erlotinib [19]; vorinostat, a histone deacetylase inhibitor, enhanced the response to carboplatin or paclitaxel in patients with advanced NSCLC [20]; MS-275, also a histone deacetylase inhibitor, enhanced the response to erlotinib in an erlotinib-resistant lung adenocarcinoma cell line [21].

Others of the 23 have not yet been investigated as cancer therapeutics (i.e., there are fewer than 20 Pubmed abstracts linking the drug to any type of cancer) and should be prioritized for further biological validation. For example, spiperone and pimozide are antipsychotics. Recently, pimozide was shown to reduce the viability of several cancer cell lines while sparing normal cells [22]. For all these new drugs that show *in vitro* anticancer activity, it will be important to determine their toxicity to normal cells.

We call this set of 23 drugs that transcriptionally reverse lung cancer gene changes and slow growth in lung cancer cell lines—TOP drugs (S2 Table); in subsequent sections, we prioritize significant drugs that have not been tested in NCI-60 by linking them to TOP drugs using a variety of metrics.

### Prioritizing drugs by structural similarity: Eleven significant drugs are highly structurally similar to TOP drugs

The Tanimoto coefficient quantifies the chemical structure similarity between two molecules [23]; here, we call two molecules structurally similar if this number exceeds 0.8. We found that eleven drugs that reverse the transcriptional changes observed in lung cancer were structurally similar to one or more drugs in TOP (Fig. 4, right; S3 Table). These drugs were not evaluated as part of the NCI-60 project; furthermore, 9 of 11 appear in fewer than 20 Pubmed abstracts concerned with cancer. These are novel candidate anticancer therapeutics identified by our computational screen. Further cell-based screens and experimental characterization would be required to determine whether these structurally similar drugs show true anticancer activity.

### Prioritizing drugs by shared target: Twenty-eight significant drugs share a protein target with one or more TOP drugs

**Fig 5:**
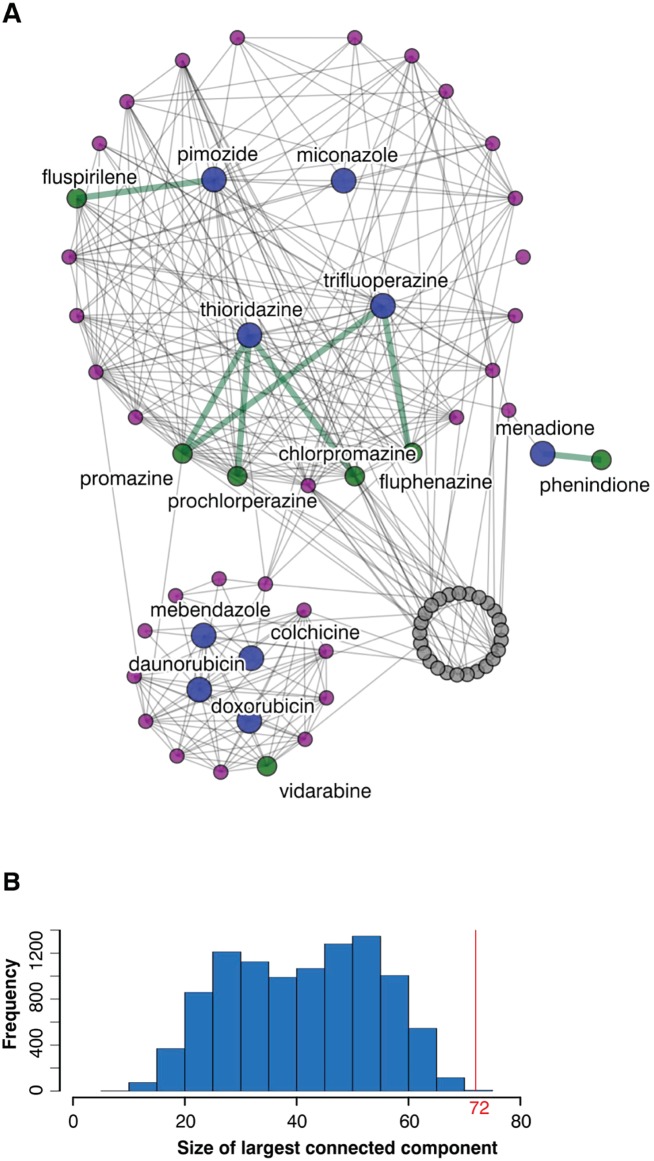
Significant drugs share many protein targets. **A**. In the drug-target network for drug candidates, two drugs are connected by an edge if they have the same protein target. Shown in colour are the drugs that slow growth in 5 or more lung cancer cell lines (blue), their immediate neighbours (purple), and the drugs that are structurally similar to them (green). Green edges indicate drug pairs that, in addition to sharing a protein target, were also found to be highly structurally similar (see Fig. 4). **B**. 83 significant drugs are represented in the drug-target network, and the largest connected component contains 72 drugs. 10,000 random draws of 83 drugs from the drug-target network resulted in smaller connected components (median size 42 drugs; P << 0.01).

We used drug-target data from DrugBank [24] and ChemBank [25] (as provided in MANTRA [26]) to construct a drug-drug interaction network on the set of CMap drugs; two drugs are linked by an edge if they share one or more protein targets (Fig. 5A). In total, 83 of the significant drugs were present in this network (the protein targets of many drugs are still unknown), including 9 TOP drugs. Thirty-eight significant drugs that were not tested in the NCI-60 collection share one or more protein targets with a TOP drug (S4 Table; Fig. 5A, purple and green nodes), indicating they may have a similar mode of action and may inhibit growth in lung cancer cell lines. However, since drug target databases do not systematically evaluate a range of drug concentrations and off-target effects, this evidence should only be considered preliminary.

Seven of these 38 drugs were also found to be structurally similar to TOP drugs (Fig. 5A, green nodes): prochlorperazine, promazine, trifluoperazine, fluspirilene, phenindione, vidarabine, and chlorpromazine. As these drugs are linked to TOP drugs by two separate lines of evidence, they are promising candidates for further experimental validation.

### Common protein targets of significant drugs

The largest connected component in the drug-target interaction network comprised 72 drugs, which is significantly larger (P << 0.01) than what would be expected by chance; random sets of 83 drugs in the drug-drug network yield largest connected components with a median size of only 42 drugs (Fig. 5B). This indicates that some gene targets are overrepresented among significant drugs; these genes may be valuable drug targets for lung cancer. We applied the hypergeometric test to each gene target of a significant drug and identified ten over-represented targets (P < 0.05; Table 2).

**Table 2:** Common protein targets of candidate drugs.

Gene	P value	Count (significant CMap drugs)	Count (all CMap drugs)
**CALM1**	5.42E-07	8	9
**PLA2G4A**	0.000288	4	4
**DRD2**	0.0006	12	34
**HTR2A**	0.000614	9	21
**SERPINA6**	0.001465	5	8
**ABCC8**	0.002252	3	3
**CYP3A3**	0.002252	3	3
**SLC6A4**	0.002321	7	16
**KCNH2**	0.002956	5	9
**SLC6A2**	0.005594	6	14
**ADRA1A**	0.008209	8	24
**ABCB1**	0.008724	5	11

The top over-represented gene is Calmodulin 1 (CALM1), a gene involved in the cell cycle and in signal transduction; it’s a target of 9 CMap drugs, and we found that 8 of these reverse the transcriptional changes seen with lung cancer. Recent research suggests that CBP501, a drug currently in Phase II clinical trials for NSCLC, may sensitize tumors to the chemotherapeutic agents bleomycin and cisplatin by inhibiting CALM1 [27]. Thus, other significant drugs that target CALM1 may also enhance the effect of chemotherapy. The 8 drugs we identified are bepridil, felodipine, flunarizine, fluphenazine, loperamide, phenoxybenzamine, pimozide, and miconazole.

The second-most overrepresented gene is PLA2G4A, whose protein product is a member of the cytosolic phospholipase A2 family. Cytosolic phospholipase A2 (cPLA2) has been previously implicated in cancer progression and metastasis. Furthermore, in a mouse model of lung cancer, the inhibition of cPLA2 activity led to delayed tumour growth [28]. There are 4 drugs targeting PLA2G4A included in the CMap collection, and all 4 significantly reverse lung cancer gene changes in our analyses: flunisolide, fluocinonide, fluorometholone, and medrysone.

### Significant drugs are broad-acting: They affect more genes than other drugs

**Fig 6:**
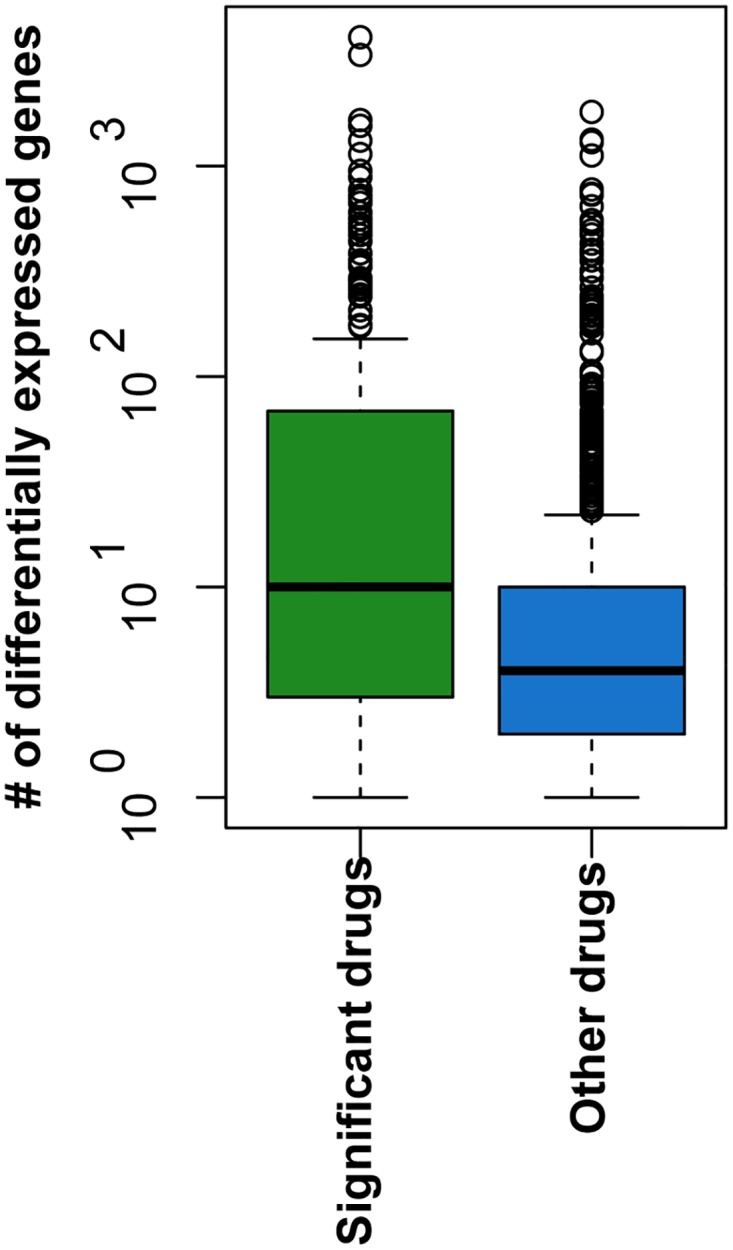
Significant drugs affect more genes than other Connectivity Map drugs. We used CMap data to calculate the number of genes that were significantly differentially regulated (P < 0.05) for each of 1,309 drugs. Drugs that we identified as reversing the gene changes seen with lung cancer affected significantly more genes than other drugs (median of 8.5 vs. 3 genes; Wilcox test P << 0.01).

We used the CMap gene expression profiles from before and after drug treatment to calculate the number of genes differentially expressed in response to a drug, for each of the 1,309 drugs in the collection (see Materials and Methods). We found that significant drugs affect a median of 8.5 genes, while other CMap drugs affect only a median of 3 (Fig. 6; Wilcox test P << 0.01).

### Many drugs are indicated for lung cancer independently of subtype

**Fig 7:**
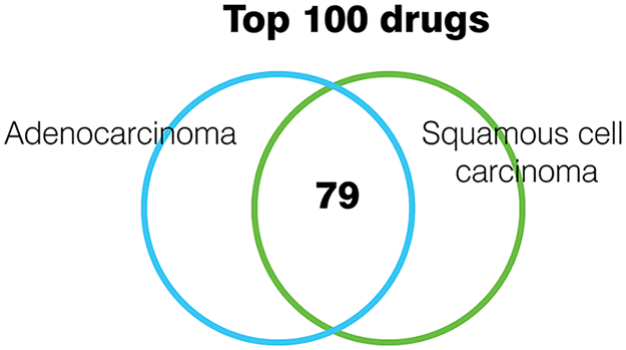
Drugs treat multiple subtypes of lung cancer. We ran CMapBatch on 10 adenocarcinoma signatures only, and on 6 squamous cell carcinoma signatures only. 79 drugs were common to the lists of top 100 drugs for both cancer subtypes.

We investigated the top drugs that revert expression changes in different lung cancer subtypes by running CMapBatch on the two largest signature subsets in our collection, adenocarcinoma (10 signatures) and squamous cell carcinoma (6 signatures). We found a very high concordance among top drugs; 79 drugs are common to the top 100 drugs lists for adenocarcinoma and squamous cell carcinoma (Fig. 7). Furthermore, all 79 drugs are significant in the full 21-signature meta-analysis (FDR < 5%). This finding is consistent with previous work showing that a common transcriptional program contributes to the molecular signature of many diverse cancers [29], and that CMap predicts similar sets of drugs for some cancers originating from different tissues [12]. Many of the current FDA approved drugs for lung cancer are also approved for other cancers, e.g. methotrexate, cisplatin, etoposide, etc [30].

### Pimozide shows robust anticancer activity *in vitro*

**Fig 8:**
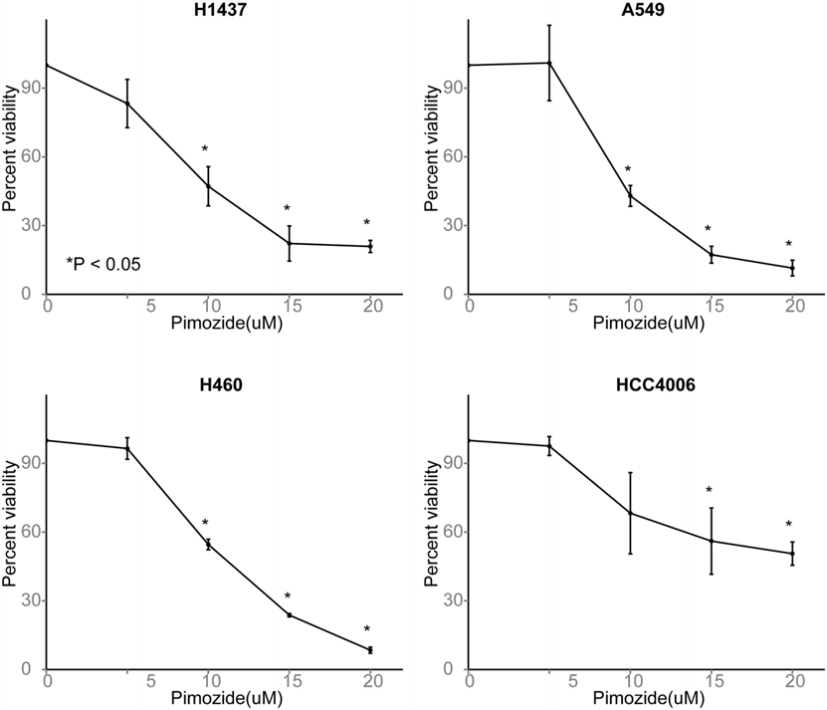
Pimozide reduces viability in four lung cancer cell lines. Results of the MTT assay in A549, HCC4006, H1437, and H4006 cell lines. Bar height indicates the mean and error bars the standard deviation of 3 biological replicates. Y-axis shows percent viability relative to untreated cells. In each cancer cell line, pimozide shows a significant cytotoxic effect. Asterisk indicates P < 0.05 (t-test).

We selected the antipsychotic drug pimozide for further *in vitro* validation. Pimozide is both a member of the set of TOP drugs and an inhibitor of CALM1, the most overrepresented protein amongst all targets of significant drugs. We conducted experiments in four lung cancer cell lines that all overexpress CALM1, A549, H460, HCC4006, and H1437 (Cancer Cell Line Encyclopedia data [31]), to test the growth slowing effects of pimozide. Using the MTT assay, we found that pimozide showed significant anticancer activity in each of the four cell lines (P ≤ 0.05; Fig. 8). This validates our computational prediction that pimozide may help treat lung cancer. Since drug-target databases predict that pimozide inhibits CALM1, we assayed CALM1 expression before and after drug treatment in A549 and H460 cells to determine whether CALM1 inhibition might mediate the anticancer activity, but found no significant difference. We also tested whether pimozide was synergistic with cisplatin using the Chou-Talalay method[32,33] in all four cell lines, but our results were negative. Our experiments confirm that pimozide shows some initial promise as a lung cancer therapeutic, but the mechanism of its anticancer activity is unknown and appears to be CALM1-independent.

## Discussion

For many diseases, including several cancers, dozens of distinct transcriptional signatures are available. We developed CMapBatch to efficiently integrate these data with the Connectivity Map to automate drug repurposing and identify stable lists of candidate therapeutics. We applied it to perform the largest *in silico* drug screen on lung cancer transcriptomes. In total, we identified 247 candidate therapeutics, and for many of these we were able to obtain additional compelling evidence from high-throughput NCI-60 data and databases of known drug targets.

CMapBatch provides a principled approach to combining drug results across multiple gene signatures of disease. Several simple extensions may be appropriate in different applications. For example, weights could be incorporated so that some studies are weighted more highly than others. Also, instead of a meta-analysis across signatures, each of which incorporates multiple patient samples, CMapBatch could be extended to a meta-analysis across all individual samples. We anticipate that CMapBatch and similar methods that can take advantage of the full set of public data on disease will help speed the discovery and development of new medicines.

## Materials and Methods

### Code and software

Code for all analyses was written in R 2.14.0. We converted gene names to HG-U133A probeset IDs for Connectivity Map analysis using the hgu133a.db (Bioconductor 2.8). The drug-target and mode of action networks were analyzed using igraph (Bioconductor 2.8) and visualized using NAViGaTOR 2.3.2 [34], and drug structures were visualized with PyMOL [35]. We calculated Tanimoto similarity for all pairs of 1,148 CMap drugs for which PubChem IDs were available using the PubChem Chemical Structure Clustering Tool [36].

### Data sources

#### Transcriptional signatures of lung cancer

We downloaded 21 gene signatures of lung cancer from CDIP version 1.0, the Cancer Data Integration Portal (http://ophid.utoronto.ca/cdip/) and Oncomine (https://www.oncomine.org/). For CDIP, we included signatures from all lung cancer vs. normal comparisons where 10 or more genes were found to be differentially up- and down-regulated. For Oncomine signatures, we sorted up- and down-regulated genes by adjusted P-value, using a threshold of FDR <= 0.05; we retained only the top 250 up-regulated and top 250 down-regulated genes.

#### Drug-response data

We downloaded rankMatrix, containing the ranks of genes in response to 6,100 drug treatments (corresponding to 1,309 unique drugs), from Connectivity Map Build 02 at http://www.broadinstitute.org/cmap/.

#### Interaction networks

We downloaded the drug-target interaction network, where two drugs share an edge if they share a physical binding partner, from MANTRA [26]. We visualized the drug target interaction network with NAViGaTOR 2.3.2 [34].

#### Lists of genes differentially regulated by CMap drugs

We downloaded lists of genes significantly up- or down-regulated by CMap drugs from [37].

### Connectivity map analysis of lung cancer signatures

#### Mapping gene names to probeset IDs

We mapped human gene IDs to Affymetrix HG-U133A IDs for connectivity map analysis following previously established protocols [3].

#### Calculating mean connectivity scores for each signature

For each lung cancer signature, mean connectivity scores for 1,309 drugs were calculated as previously described [3] and converted to ranks.

### Meta-analysis of drug-response data

We have made the CMapBatch meta-analysis workflow available as an R script from http://www.cs.utoronto.ca/~juris/data/cmapbatch.

#### Combining ranked lists of drugs to construct a consensus ranked list

We adapted the Rank Product method [15] to identify drugs that consistently reverse the transcriptional changes seen within lung cancer across a large collection of signatures. Previous authors have converted CMap scores to drug ranks for downstream analyses (e.g., [26]), but no one has previously combined ranks across multiple signatures in a meta-analysis. For each drug, we calculated the product of its ranks in all lung cancer signatures.

#### Identifying drugs with significantly small rank products

We randomly permuted the assignment of KS scores to drugs for the 6,100 instances (drug treatments), recalculated mean scores and drug ranks for 1,309 drugs in each signature, and re-calculated randomized rank products 10,000 times. We used this background distribution to calculate p-values and estimate false discovery rates.

### NCI-60 analysis of significant drugs

We restricted our analyses to the NCI-60 GI50 (50% growth inhibition) data and to those lung cancer cell lines where at least 100 Connectivity Map drugs were tested (there were nine of these, all NSCLC: NCI-H23, NCI-H522, A549/ATCC, EKVX, NCI-H226, NCI-H322M, NCI-H460, HOP-62, HOP-92). As different GI50 thresholds were used to denote minimal activity in response to a drug for different concentration ranges, we filtered the data to make results comparable across drugs. We retained only those entries with an LCONC (maximum log10 concentration) of-4 and where the drug concentration was measured in units of molarity.

### Experimental validation of drug candidates

#### Cell lines and drug treatments

NSCLC A549, H460, HCC4006, and H1437 cell lines were cultured in RPMI 1640 with 10% fetal bovine serum (FBS), and 1% penicillin/streptomycin. Pimozide was purchased from Sigma (St. Louis, USA). Pimozide was resuspended in DMSO.

#### IC50 determination and cytotoxicity assays

Cells were plated in 96 well plates at 2000–5000 cells per well (depending on the cell line). 24 hours after seeding, cells were incubated in the presence of different concentrations of pimozide. After 48 hours they were treated with MTT (300 ng/ul) (3-[4, 5-dimethylthiazol-2-yl]-2, 5-diphenyl tetrazolium bromide) at 37°C for 2 h. The converted formazan crystals were solubilized with 100 ul of DMSO and the absorbance was read at 540 nm in a monochromator reader (Tecan, Switzerland). [38]. Assays were performed in triplicate. The IC50 values obtained were used to treat cells for 48 hours for all subsequent assays. P-values were calculated with the Student’s t-test.

## Supporting Information

S1 Table 247 significant drugs consistently reverse lung cancer gene changes in rank product meta-analysis. (XLS)

S2 Table 23 TOP drugs are significant in meta-analysis and inhibit growth in a majority of NCI60 lung cancer cell lines. (XLS)

S3 Table 11 significant drugs are structurally similar to one or more TOP drugs. (XLS)

S4 Table 38 significant drugs share one or more protein targets with a TOP drug. (XLS)

S1 Fig CMapBatch produces more stable lists of drugs than individual gene signatures, using multiple cut-offs. (TIF)
