# Supplementary figures and images for: Prioritizing Therapeutics for Lung Cancer: An Integrative Meta-analysis of Cancer Gene Signatures and Chemogenomic Data
Source: PLoS Comput Biol. 2015 Mar 18;11(3):e1004068. doi: 10.1371/journal.pcbi.1004068 (PMC4364883; doi:10.1371/journal.pcbi.1004068)

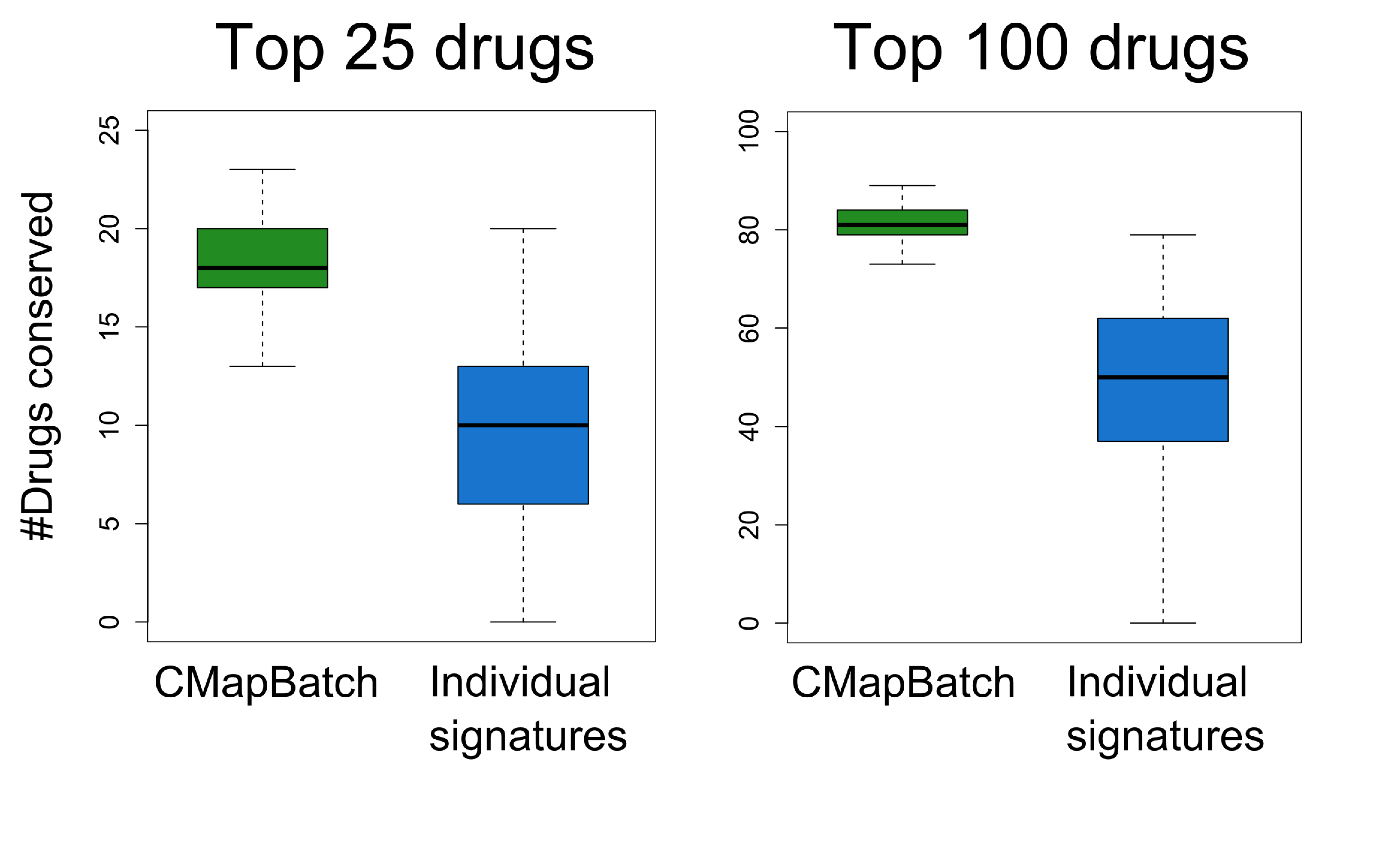

Supplement: S1 Fig — (TIF) [file pcbi.1004068.s005.tif]
